# A novel lncRNA YIL163C enhances genomic stability and antifungal resistance via the DNA damage response in *Saccharomyces cerevisiae*

**DOI:** 10.3389/fmicb.2025.1571797

**Published:** 2025-05-01

**Authors:** Xueting Wang, Xuemei Li, Duoyun Li, Yiying Zhang, Bing Bai, Bao Chai, Zewen Wen

**Affiliations:** ^1^Department of Dermatology, Shenzhen Nanshan People’s Hospital, Affiliated Nanshan Hospital of Shenzhen University, Shenzhen, China; ^2^Guangdong Key Laboratory for New Technology Research of Vegetables, Vegetable Research Institute, Guangdong Academy of Agricultural Sciences, Guangzhou, China; ^3^Department of Infectious Diseases and Shenzhen Key Laboratory for Endogenous Infections, Shenzhen Nanshan People’s Hospital, Affiliated Nanshan Hospital of Shenzhen University, Shenzhen, China

**Keywords:** lncRNA, DNA damage response, 5-fluorocytosine, phosphoproteome, YIL163C

## Abstract

**Introduction:**

Long non-coding RNAs (lncRNAs) are increasingly recognized as key regulators in cellular processes, including the DNA damage response (DDR). In *Saccharomyces cerevisiae*, DDR is critical for maintaining genomic integrity under stress, mediated by proteins like Mec1 and Rad53. However, the involvement of lncRNAs in DDR pathways, remains largely unexplored. This study investigates the function of a novel lncRNA, YIL163C, in promoting cell survival and genomic stability under DNA damage conditions.

**Methods:**

Genetic suppressor screening was employed to assess the role of YIL163C in rescuing lethality in *mec1Δ sml1Δ* and *rad53Δ sml1Δ* exposed to DNA damage. Proteomic and phosphoproteomic analyses were conducted to evaluate changes in protein abundance and phosphorylation states. The impact of YIL163C on DDR and antifungal drug tolerance, specifically to 5-fluorocytosine, was also examined.

**Results:**

Overexpression of YIL163C was found to rescue lethality in *mec1Δ sml1Δ* and *rad53Δ sml1Δ* under DNA damage conditions. Proteomic analyses revealed that YIL163C modulates pathways related to DNA replication, ER stress response, and ribosome biogenesis, enhancing cellular resilience to HU-induced stress. Additionally, YIL163C reduced sensitivity to 5-fluorocytosine, indicating a role in antifungal drug tolerance. Phosphoproteomic data suggested YIL163C influences phosphorylation states, potentially acting downstream of the Mec1-Rad53 signaling pathway.

**Conclusion:**

This study provides new insights into the regulatory mechanisms of lncRNAs in DDR, with broader implications for antifungal therapy and genomic stability research, emphasizing the role of lncRNAs in stress responses beyond traditional protein-centric mechanisms.

## 1 Introduction

Genomic integrity is critical for the accurate transmission of genetic information across generations, ensuring that cells maintain their viability and function. Throughout their lifespan, cells are constantly exposed to various internal and external stressors, such as replication errors, reactive oxygen species (ROS), radiation, chemicals, and chemotherapeutic agents. These stressors can cause significant DNA damage, which, if left unchecked, compromises genomic stability (Ciccia and Elledge, 2010). DNA damage may manifest in several forms, including base modifications, mismatches, and single- or double-strand breaks (DSBs). Accumulation of such damage can lead to severe outcomes, including direct cell death, apoptosis, or abnormal cell proliferation, which is often associated with cancer development. The eukaryotic cells have evolved a highly conserved mechanism known as the DDR. The DDR system acts as a cellular safeguard that detects and repairs DNA damage while halting the cell cycle to provide the necessary time for repairs (Li et al., 2016). The DDR encompasses a complex network of pathways, including the DNA replication checkpoint (DRC), chromatin remodeling, and DNA repair pathways. These checkpoints play an essential role in coordinating the cell cycle and DNA repair, ensuring the stability of the genome and cell survival under genotoxic stress (Lu et al., 2023). Defects in the human DDR underpin several genomic instability syndromes and can contribute to tumorigenesis (Huang and Zhou, 2021; Jackson and Bartek, 2009; Shimizu et al., 2014). Therefore, targeting DDR in cancer have provide significant opportunities for DDR-based therapies in the future (Cheng et al., 2022; Groelly et al., 2023; O'Connor, 2015).

The budding yeast, *S. cerevisiae*, has been a powerful model organism for studying the mechanisms of DDR, largely due to its simple genetic framework and highly conserved DDR pathways. In yeast, the sensor kinase Mec1 (the homolog of human ATR) responds to DNA damage and replication stress (Friedel et al., 2009). Mec1 initiates the DDR by phosphorylating and activating the checkpoint kinase Rad53, which in turn orchestrates a cascade of downstream events critical for cell survival. Activated Rad53 phosphorylates multiple effector proteins, including Dun1, which regulates genes responsible for DNA repair, ribonucleotide reduction, and replication fork stabilization (Sanford et al., 2021). One of the major roles of the Mec1-Rad53 pathway is to regulate ribonucleotide reductase (RNR), a key enzyme involved in maintaining the balance of deoxyribonucleotide triphosphate (dNTP) pools essential for DNA synthesis and repair (Chabes et al., 2003). Proper regulation of RNR is vital, as imbalanced dNTP levels can lead instability of genomic instability. Mec1 and Rad53 enhance RNR activity by promoting the degradation of Sml1, a small protein that inhibits RNR. The removal of Sml1 increases dNTP levels, facilitating DNA repair and replication under conditions of stress (Zhao and Rothstein, 2002).

While the DDR has traditionally was a protein-centric process, recent studies have uncovered the involvement of long non-coding RNAs (lncRNAs) in various cellular functions, including the regulation of DDR (Su et al., 2018). Traditional studies define lncRNAs as non-protein-coding transcripts longer than 200 nucleotides. However, recent research challenges the traditional notion that lncRNAs are strictly noncoding. Emerging evidence reveals that some lncRNAs harbor the potential to be translated, including in *S. cerevisiae* (Andjus et al., 2024; Smith et al., 2014). Ribosome profiling and other advanced methodologies have provided direct evidence of lncRNA translation or association with ribosomes. Although research on lncRNAs is still in its early stages, they have been shown to play roles in gene transcription, post-transcriptional regulation, and direct DNA repair (Wan et al., 2014). LncRNAs have been found to play important roles in DDR and repair, functioning through various mechanisms. Firstly, lncRNAs can regulate gene transcription or degradation during the DDR/repair process through different mechanisms. For instance, the DNA damage-induced transcript lncRNA-gadd7 binds the TAR DNA-binding protein (TDP-43) and disrupts its interaction with Cdk6 mRNA, leading to the degradation of Cdk6 mRNA and regulation of the G1/S checkpoint after DNA damage (Liu et al., 2012). Additionally, lncRNAs are involved in maintaining genomic stability by modulating DDR processes (Chu et al., 2017; Cusanelli and Chartrand, 2015; Lee et al., 2016; Porro et al., 2014). Moreover, lncRNAs can directly participate in DNA repair. For example, lncRNA DDSR1 is activated through transcription in response to DSB-inducing agents, in an ATM-dependent manner. DDSR1 interacts with BRCA1 and hnRNPUL1, recruiting hnRNPUL1 to the site of damage to facilitate DNA end resection, thereby promoting DDR signaling and homologous recombination (HR) repair (Sharma et al., 2015).

Despite the growing interest in lncRNAs, their involvement in the DRC remains largely unexplored. In this study, we identified a novel lncRNA, which we term YIL163C, through genetic suppressor screening. This lncRNA was found to rescue the lethality of yeast cells lacking Mec1 or Rad53, key components of the DDR pathway. Our findings suggest that YIL163C may functions as a novel effector downstream of the Mec1-Rad53 signaling axis, promoting cell survival during replication stress. The primary aim of this study is to characterize the function of YIL163C in the context of the yeast DDR pathway, particularly in response to replication stress induced by HU. Using a combination of proteomic and phosphoproteomic analyses, we explore how YIL163C modulates protein abundance and phosphorylation during DDR. Additionally, the role of YIL163C in modulating yeast cell susceptibility to antifungal drugs, including 5-fluorocytosine was investigated. Our findings provide novel insights into the regulatory mechanisms of DDR, highlighting the potential of lncRNAs as regulators of genome stability and therapeutic targets.

## 2 Methods

### 2.1 Yeast strains, plasmids, and manipulations

The *S. cerevisiae* strains used in this study include BY4741 and its respective mutants. A detailed list of the yeast strains and plasmids can be found in the Table 1. Yeast was routinely cultured in YPD medium (1% yeast extract, 2% peptone, and 2% glucose) at 30°C. Strains harboring pRS316 backbone plasmids were maintained in synthetic complete dropout (SC) medium lacking uracil. In both YPD and SC media, glucose (2%) served as the primary carbon source.

**Table 1:** Yeast strains used in this study.

Strain	Relevant genotype	Reference or source
BY4741	MATa his3Δ1 leu2Δ0 met15Δ0 ura3Δ0 lys2Δ0	Brachmann et al. (1998)
*mec1Δ sml1Δ*	*BY4741 mec1Δ::G418 sml1Δ::LEU2*	This study
*rad53Δ sml1Δ*	*BY4741 rad53Δ::G418 sml1Δ::LEU2*	This study
*YIL163CΔ*	*BY4741 YIL163CΔ::NatMX*	This study
*mec1Δ sml1Δ-EV*	*BY4741 mec1Δ::G418 sml1Δ::LEU2* with empty pRS316	This study
*mec1Δ sml1Δ YIL163CΔ*	*BY4741 mec1Δ::G418 sml1Δ::LEU2 YIL163CΔ::NatMX*	This study
*mec1Δ sml1Δ YIL163C-OE*	*BY4741 mec1Δ::G418 sml1Δ::LEU2* with pRS316-YIL163C	This study
*mec1Δ sml1Δ YIL163C_TAG_-OE*	*BY4741 mec1Δ::G418 sml1Δ::LEU2* with pRS316-YIL163C_TAG_	This study
*mec1Δ sml1Δ YIL163C 400 nt-OE*	*BY4741 mec1Δ::G418 sml1Δ::LEU2* with pRS316-YIL163C 400 nt	This study
*mec1Δ sml1Δ YIL163C 400 nt + terminator-OE 1# 2# 3#*	*BY4741 mec1Δ::G418 sml1Δ::LEU2* with pRS316-YIL163C 400 nt + terminator	This study
*mec1Δ sml1Δ RNR3 pro-YIL163C-OE*	*BY4741 mec1Δ::G418 sml1Δ::LEU2* with pRS316- *RNR3 pro-YIL163C*	This study
*rad53Δ sml1Δ-EV*	*BY4741 rad53Δ::G418 sml1Δ::LEU2* with empty pRS316	This study
*rad53Δ sml1Δ YIL163C-OE*	*BY4741 rad53Δ::G418 sml1Δ::LEU2* with pRS316-YIL163C	This study
*rad53Δ sml1Δ RNR3 pro-YIL163C-OE*	*BY4741 rad53Δ::G418 sml1Δ::LEU2* with pRS316- *RNR3 pro-YIL163C*	This study

To construct the overexpression vector of YIL163C, the full-length fragment of YIL163C was amplified using primers *YIL163C-SalI-F*/*YIL163C-BamHI-R* and cloned into pRS316 vector to yield pRS316- YIL163C (Table 2). Other overexpressed lines are also amplified by PCR using the corresponding primers and subsequently cloned into the pRS316 vector.

**Table 2:** Oligonucleotide primers used in this study.

Primer	Sequence (5′-3′)
*YIL163C-SalI-F*	ACGCGTCGACTCAGGAGTGTTATAAGTCC
*YIL163C-BamHI-R*	CGCGGATCCTAGTAAACAGGGAGATACCG
*3′-check-R-3 (400-420)*	TACCAAAGGCGTGCCTTTGT
*3′-check-R-6 (335-354)*	TTACTCTGAACAGGAATAAA
*3′-check-R-7 (355-374)*	CATTATTCCCCGCATTTTTA
*5′-check-F-5 (1-20)*	ATGTTTCTTTTCAGGAGGAA
*5′-check-F-6 (-20-1)*	AATAATACATATCTATTTAT
*3′-check-R-7 (375-394)*	CGATCCATTATGAGGGCTTC
*3′-check-R-8 (390-410)*	GTGCCTTTGTTGAACTCGATC
*YIL163C (with N.P) -SalI-F*	GGTACCGGGCCCCCCCTCGAGGTCGACTTCAGGAGTGTTATAAGTCC
*YIL163C (400 bp) BamHI-R*	GTGGCGGCCGCTCTAGAACTAGTGGATTGAACTCGATCCATTATGAG
*ADH1 terminator-SpeI-F*	CCCTCATAATGG ATCGAGTTCAATCCAGCGAATTTCTTATGATTTATG
*ADH1 terminator-SacI-R*	GTAATACGACTCACTATAGGGCGAATT CCGGTAGAGGTGTGGTCAATA
*RNR3 pro-F-KpnI*	GGGGTACCAGCACATAAAAAATCAGCAC
*RNR3 pro-R-XhoI*	CCGCTCGAGTTGTGTGGGAGTATTTGATT
*YIL163C (ATG)-SalI-F (ATG)*	ACGCGTCGACATGTTTCTTT TCAGGAGGAA
*YIL163C (with N.T) BamHI-R*	CGGGATCC CCTAGTAAACAGGGAGATACCG

To determine the length of the YIL163C transcript, total RNA is extracted, and reverse transcription (RT) is performed using random primers to synthesize cDNA. PCR amplification is then conducted using the cDNA as a template. To identify the transcript length of YIL163C, the PCR primers are progressively shortened (Table 2), and the resulting products are analyzed to establish the precise transcript size.

### 2.2 Spot assay

Equal amounts of starting overnight yeast cultures (OD_600_ = 0.4) were subjected to five-fold serial dilutions, followed by spotting onto appropriate medium containing indicated concentrations of HU or other drugs. Plates were then incubated at specific temperatures (30°C) for 48 h, or as otherwise specified, and subsequently photographed. For drug susceptibility analysis, the indicated *S. cerevisiae* strains were spotted in 5-fold dilutions starting at an OD600 of 0.1 on untreated YPD plates or plates containing 4 μg/mL ketoconazole, 4 μg/mL amphotericin B, 16 μg/mL 5-fluorocytosine (Sigma-Aldrich, St. Louis, MO). Plates were grown at 30° for 2 days before photographed.

### 2.3 Protein sample preparation and extraction

*S. cerevisiae* strains *mec1Δ sml1Δ* with pRS316 empty vector (*mec1Δ sml1Δ-EV*) and *mec1Δ sml1Δ*-YIL163C-OE were grown overnight at 30°C and then inoculated into fresh SC-URA medium, shaking at 200 rpm until reaching the logarithmic phase. HU was then added to a final concentration of 10 mM, and the cultures were further shaken for 2 h. Cells were collected by centrifugation at 4°C and washed three times with cold lysis buffer (10 mM Tris/HCl). The cells were then resuspended in lysis buffer with the addition of a cOmplete Protease Inhibitor Cocktail (Roche, Basel, Switzerland). For lysis, cells underwent three rounds of homogenization using 0.1 mm glass beads, with each round followed by 1-min incubation on ice. Dithiothreitol (DTT) was added to the lysate to a final concentration of 10 mM. The samples were centrifuged at 20,000 × g for 20 min, and the supernatant was mixed with ice-cold acetone/30 mM DTT. This step was repeated twice, after which the supernatant was pooled and precipitated overnight at −20°C. After centrifugation, the protein pellet was resuspended in digestion buffer [100 mM triethylammonium bicarbonate (TEAB), 0.05% w/v sodium dodecyl sulfate (SDS)] to a final concentration of 1 mg/mL (total protein concentration was determined using the BCA assay). Proteins were digested overnight with trypsin (Promega, Madison, WI) at a ratio of 1:50 (w/w) at 37°C.

### 2.4 LC–MS/MS and quantitative proteomics analysis

The samples were resuspended in 0.1% formic acid solution. Liquid chromatography was performed using an UltiMate 3000 RSLC nano system coupled with a C18 pre-column (100 μm × 20 mm, Acclaim PepMap 100 C18, 3 μm) and a C18 column (75 μm × 250 mm, Acclaim PepMap RSLC, 2 μm). Mobile phases A and B consisted of 0.1% formic acid and 80% acetonitrile in 0.1% formic acid, respectively. The columns were connected to a Q Exactive Plus mass spectrometer (Thermo Scientific, Waltham, MA) equipped with a nano-electrospray ionization (NSI) interface. MS1 scans were acquired over a mass range of 300–1,500 m/z with a resolution of 70,000, and corresponding MS2 spectra were acquired with a resolution of 17,500, with a maximum collection time of 50 ms.

The raw LC–MS/MS data were processed using Proteome Discoverer 2.4 and Sequest HT for protein identification and quantification against the *S. cerevisiae* (strain ATCC 204508/S288c) Uniprot proteome database. Proteins were considered up-regulated or down-regulated if they met a 2-fold cut off and a *p*-value of < 0.05, based on at least two technical replicates. Differentially expressed proteins were annotated for GO, including biological processes, cellular components, molecular functions, and analyzed for KEGG pathways. PPI networks were analyzed using the web-based tool STING.

### 2.5 Four-dimensional independent data acquisition (4D-DIA) phosphorylation proteomics

Protein digestion was performed using the filter-aided sample preparation (FASP) method, as described in previous (Wiśniewski et al., 2009). Briefly, proteins were dissolved in 8 M urea, reduced with DTT, and alkylated with iodoacetamide. The samples were then applied to a 10 kDa molecular weight cut off filter. Washing steps were performed to remove excess impurities, and trypsin was introduced for enzymatic digestion. The flow-through was collected and lyophilized.

For phosphopeptide enrichment, a High-Select Fe-NTA Phosphopeptide Enrichment Kit (Thermo Fisher Scientific) was used following the manufacturer’s instructions. In brief, peptides were resuspended in 200 μL of binding/wash buffer and loaded onto an equilibrated spin column. The resin was mixed with the sample by gently tapping the column, and the mixture was incubated for 30 min. The column was centrifuged at 1,000 g for 30 s, and the flow-through was discarded. The column was then washed three times with 200 μL of binding/wash buffer and centrifuged at 1,000 g for 30 s, followed by a single wash with 200 μL of LC–MS-grade water. Phosphopeptides were eluted by adding 100 μL of elution buffer and centrifuging at 1,000 g for 30 s, repeated twice. The phosphopeptides were dried and subjected to LC–MS/MS analysis.

### 2.6 Mass spectrometry analysis and data processing for phosphorylation proteomics

Following centrifugation and drying, the digested peptide samples were reconstituted in Nano-LC mobile phase A (0.1% formic acid in water) and loaded for online LC–MS analysis. A 2 μL aliquot of the reconstituted sample was injected onto a trapping column (AURORA PARID75, 75 μm × 5 cm, C18, 1.7 μm, 100 Å). Chromatographic separation was performed on an Ultimate 3000 nano-flow liquid chromatography system (Thermo Fisher Scientific, United States). After desalting and retention on the trapping column, peptides were separated using an analytical C18 reversed-phase column (AURORA Ultimate, 75 μm × 25 cm, C18, 1.7 μm, 100 Å). The gradient used for separation increased mobile phase B (80% acetonitrile with 0.1% formic acid) from 5 to 38% over 60 min. Mass spectrometry was performed on a timsTOF Pro system (Bruker, Germany) coupled with a Nano Flex ion source (Thermo Fisher Scientific, United States) in PASEF (Parallel Accumulation–Serial Fragmentation) mode for DDA (data-dependent acquisition). The scan range was set to 100–1,700 m/z with 10 PASEF cycles. TIMS was configured with a 100 ms accumulation time (100% duty cycle) and a ramp rate of 9.43 Hz, resulting in a total cycle time of 1.17 s. A linear precursor targeting mode was applied, with a target intensity of 15,000 and a threshold of 5,000. Dynamic exclusion was set to 0.4 min, and collision energy was maintained at default settings, with 1.60/K0 [*Vs*/cm^2^] at 59 eV and 0.60/K0 [*Vs*/cm^2^] at 20 eV. Isolation widths were set to 2 m/z for precursors <700 m/z and 3 m/z for precursors >800 m/z. The TIMS range was initially set to 0.6–1.60/K0 [*Vs*/cm^2^].

### 2.7 Bioinformatics methods

The raw spectral data files acquired from mass spectrometry were processed and analyzed using Spectronaut 18 software in direct DIA mode. The UniProt *Mus musculus* reference protein database was used for data searching. The search parameters followed the default settings for the Phospho PTM Workflow, configured as follows: trypsin was used as the digestion enzyme, allowing up to two missed cleavages, with peptide lengths set between 7 and 52 amino acids. Carbamidomethylation of cysteine was selected as a fixed modification. Variable modifications included N-terminal acetylation, methionine oxidation, and phosphorylation on serine, threonine, and tyrosine residues (S/T/Y). The mass tolerance was dynamically adjusted during data analysis. The false discovery rate (FDR) was controlled at 1.0% at the PSM, peptide, and protein group levels.

### 2.8 Quantitative PCR

The *mec1Δ sml1Δ-EV* and *mec1Δ sml1Δ*-YIL163C-OE strains were cultured overnight at 30°C and subsequently inoculated into fresh SC-URA medium with continuous shaking at 200 rpm until reaching logarithmic growth phase. Cultures were then treated with HU at a final concentration of 10 mM and incubated for an additional 2 h with shaking. Total RNA was isolated using the Yeast Total RNA Rapid Extraction Kit (B518627-0050, Sangon Biotech, Shanghai), followed by cDNA synthesis using the PrimeScript RT Reagent Kit (Takara Bio). Quantitative PCR (qPCR) was performed on QuantStudio 5, with the *Actin* gene serving as an internal control. The expression levels of target genes in *mec1Δ sml1Δ*-EV were normalized to 1 for comparative analysis. The primer sequences were listed in Table 3.

**Table 3:** The primer sequences used for qPCR.

Primer	Sequence (5′-3′)
*Actin-F*	GATGTCGATGTCCGTAAGG
*Actin-R*	CAAGATAGAACCACCAATCCA
*UBX4-F*	AGCCTTCGTCAGTTAGATTG
*UBX4-R*	AGTTCGTGAGGTGGTAGT
*RVB2-F*	GACAGAATTGATTGAAGGTGAAG
*RVB2-R*	AATAACATCGCCAGCCAATA
*RTT106-F*	TCAGCCATCTATTCTACAGGA
*RTT106-R*	ATCGTCTTCTTCATCATCTTCTT
*HHO1-F*	CCAAGAAGAAATCTCCAGAAGTA
*HHO1-R*	AATGAAGAAGGCGAAGAGG
*IGO2-F*	GCTGTCACCACAAGAACT
*IGO2-R*	TAGGATTGGTCACTGGTAGAT
*UTP4-F*	ATCACCGCTGTTCATATCAATA
*UTP4-R*	TAATACGCTTTCGCTATCTTCA
*PIL1-F*	AGGTGTCTTGATCTATGAAGTTAG
*PIL1-R*	ATCTTGTCCTTACGGTCTCTA
*NHP2-F*	TGTGTTGCCATTCGCTAA
*NHP2-R*	ACTGGGATGTGGGAAATAAC
*PRI1-F*	CCAAGCAAGACTCTCACAT
*PRI1-R*	CCGTAGCAGGATGAATACAA
*ADH2-F*	ACTGTTGTCTTGGTTGGTT
*ADH2-R*	TCTAAGGCTTCTCTGGTATCA
*UBX5-F*	GACGGGAACTTGAACACA
*UBX5-R*	GCATCGGCATCTGAATCT
*CDC34-F*	CGAATAAGGATATGGCGGATAA
*CDC34-R*	CGTCATCATCGTCATCATCT
*PTC2-F*	CCGATAACGACGATCCAAT
*PTC2-R*	GTTGCTGTCTGTCTTCACT
*TAF14-F*	CGTTATCCAGATTCCTCTCAAT
*TAF14-R*	CAGTTGTGTTCGTGGTAGT
*GCD11-F*	TATGGATGCTGCGTTACTG
*GCD11-R*	CTCTGATGAACTTCAAGATGGA
*SHP1-F*	GAGAGGAAGCACATTGGAA
*SHP1-R*	CTTGGTTGGTTCAGGAGAG
*ZPR1-F*	GTGAGAACCGACGAACAA
*ZPR1-R*	TGAGCCAACTTTGACAGATT
*PCC1-F*	GACAAGCGACCATAGCAA
*PCC1-R*	CGAAGCACCCTATCATCAAT
*UBC13-F*	GGCATTACAGCAGAACCA
*UBC13-R*	AGTCGTCAGGCAGATATAATTC
*CKB1-F*	AAGAATTGGAAGAGTATGTGGAA
*CKB1-R*	CCGTGGATGGATATTGTCTC
*SMI1-F*	CGGTGATGGTGAATTGGT
*SMI1-R*	GCAAGGATGTGTCAGGTT
*SMT3-F*	CAGGGTAAGGAAATGGACTC
*SMT3-R*	GCACCACCAATCTGTTCT
*UBC1-F*	CACTATGGACGAGGTTATACG
*UBC1-R*	GCTGTGTTGTTGTCATTGG
*SKP1-F*	GACGAAGATGACGACGATT
*SKP1-R*	GGCTTGATGTTGAGGTAGTT

## 3 Results

### 3.1 Identification of YIL163C as a novel regulator of the DRC in *Saccharomyces cerevisiae*

**Figure 1:**
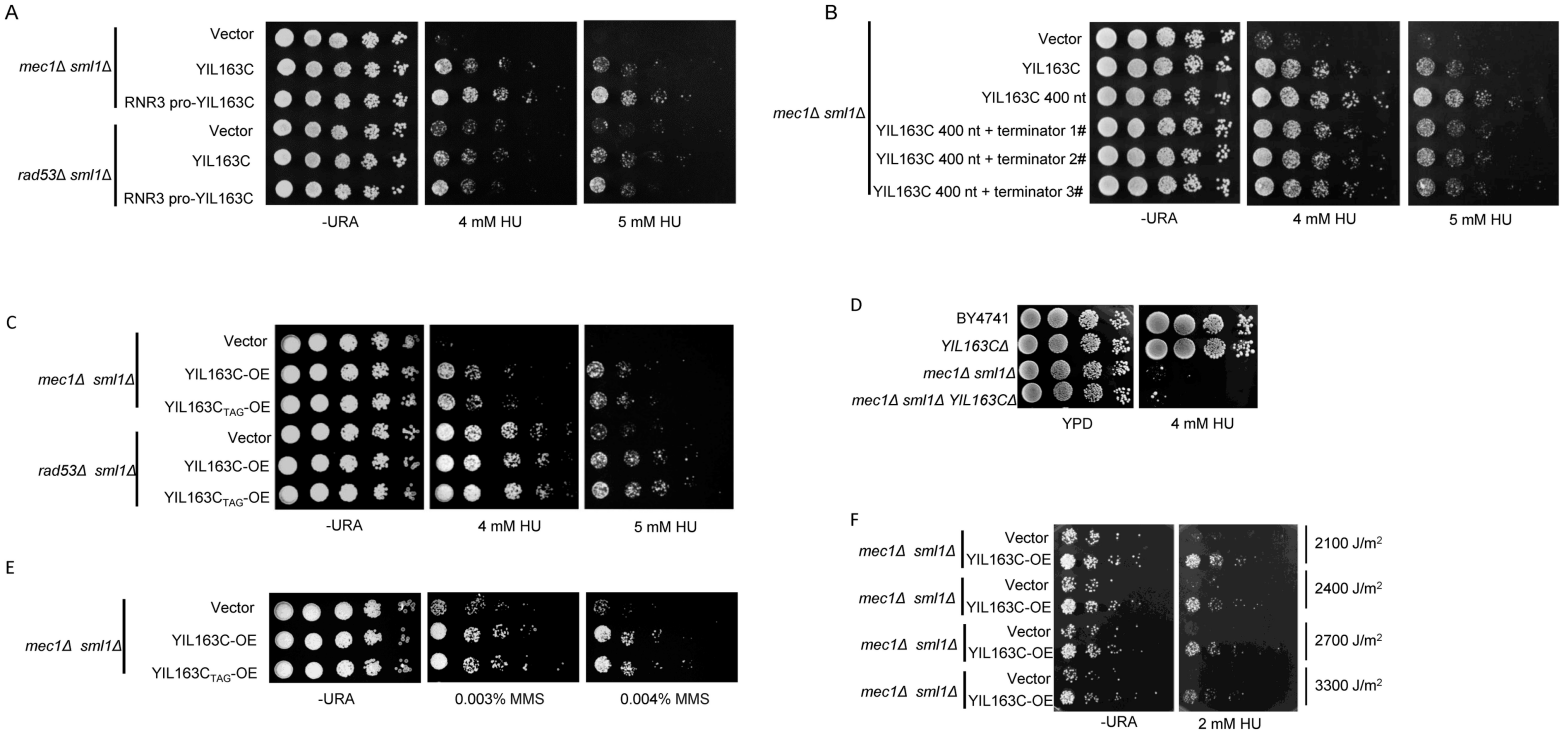
YIL163C has been identified as a novel checkpoint pathway regulator downstream of the Mec1-Rad53. **(A)** The effect of replacing the native promoter of YIL163C with the *RNR3* promoter on the HU hypersensitivity phenotype in YIL163C reconstitution in *mec1Δ sml1Δ* and *rad53Δ sml1Δ* strains. **(B)** The effect of replacing or deleting the terminator region of YIL163C on the HU hypersensitivity phenotype in YIL163C reconstitution in *mec1Δ sml1Δ*. **(C)** Gradient dilution phenotype analysis was performed to assess the impact of YIL163C overexpression on the growth phenotype of *mec1Δ sml1Δ* and *rad53Δ sml1Δ* strains. **(D)** Deletion of YIL163C does not affect yeast sensitivity to HU. Five-fold serial dilutions of the indicated strains were spotted onto SC-URA or YPD plates and incubated at 30°C for 48 h. **(E)** The impact of YIL163C overexpression on MMS-induced DDR in *mec1Δ sml1Δ*. **(F)** The effect of YIL163C overexpression on UV-induced DDR phenotypes was evaluated in *mec1Δ sml1Δ*across varying radiation doses.

To identify positive regulators of the DDR, we performed a dosage suppressor screen using the *mec1Δ sml1Δ* mutant in *S. cerevisiae*. A yeast genomic DNA library was cloned into a low-copy yeast plasmid and introduced into the *mec1Δ sml1Δ* strain. The *mec1Δ sml1Δ* cells were hypersensitive to even low doses of HU (4 mM) compared to wild-type (WT) *S. cerevisiae* (Zhao et al., 2000). By selecting and sequencing plasmids that enabled the *mec1Δ sml1Δ* cells to resist 4 mM HU, a fragment including whole YIL163C as a potential suppressor was identified. By gradually shortening the PCR primers, the transcript of YIL163C from the ATG start codon was determined to 400 bp (Supplementary Figure S1). To determine whether YIL163C is responsible for the suppression of the DDR hypersensitive phenotype in *mec1Δ sml1Δ*, the native promoter of YIL163C (including the SUC2 fragment) was replaced with the promoter of RNR3, which is induced by HU. This replacement enhanced the suppression effect (Figure 1A; Supplementary Figure S2). Furthermore, the sequence downstream of the YIL163C was truncated and added an ADH1 terminator, observing the same suppression effect (Figure 1B). These results demonstrate that YIL163C alone is sufficient to rescue the DDR hypersensitive phenotype. Based on Ribo-Seq data, YIL163C has been annotated as a protein-coding gene (Brar et al., 2012). However, when the sole start codon of YIL163C was mutated to TAG (YIL163C_TAG_), it still rescued the DDR hypersensitive phenotype of *mec1Δ sml1Δ* and *rad53Δ sml1Δ*, indicating that YIL163C functions in its RNA form (Figure 1C). Tetrad analysis demonstrated that overexpression of YIL163C in either *mec1* or *rad53* knockout strains effectively rescued the lethal phenotypes caused by the deletion of *mec1* or *rad53* (Supplementary Figure S3). However, we found that deletion of YIL163C in either the *BY4741* or *mec1Δ sml1Δ* background had no effect on sensitivity to HU (Figure 1D), while the *mec1Δ sml1Δ* and *mec1Δ sml1Δ YIL163CΔ* strains exhibit normal growth in the absence of HU. Furthermore, beyond the HU-mediated DDR, the responses of YIL163C to other DDR-inducing agents including ultraviolet (UV) radiation and methyl methyl methane sulfonate (MMS) was evaluated. The results demonstrated that overexpression of YIL163C in the *mec1Δ sml1Δ* background partially rescued the hypersensitivity of *mec1Δ sml1Δ* to either MMS or UV treatment (Figures 1E,F).

### 3.2 Proteomic changes induced by YIL163C overexpression in *mec1Δ sml1Δ* cells under replication stress

**Figure 2:**
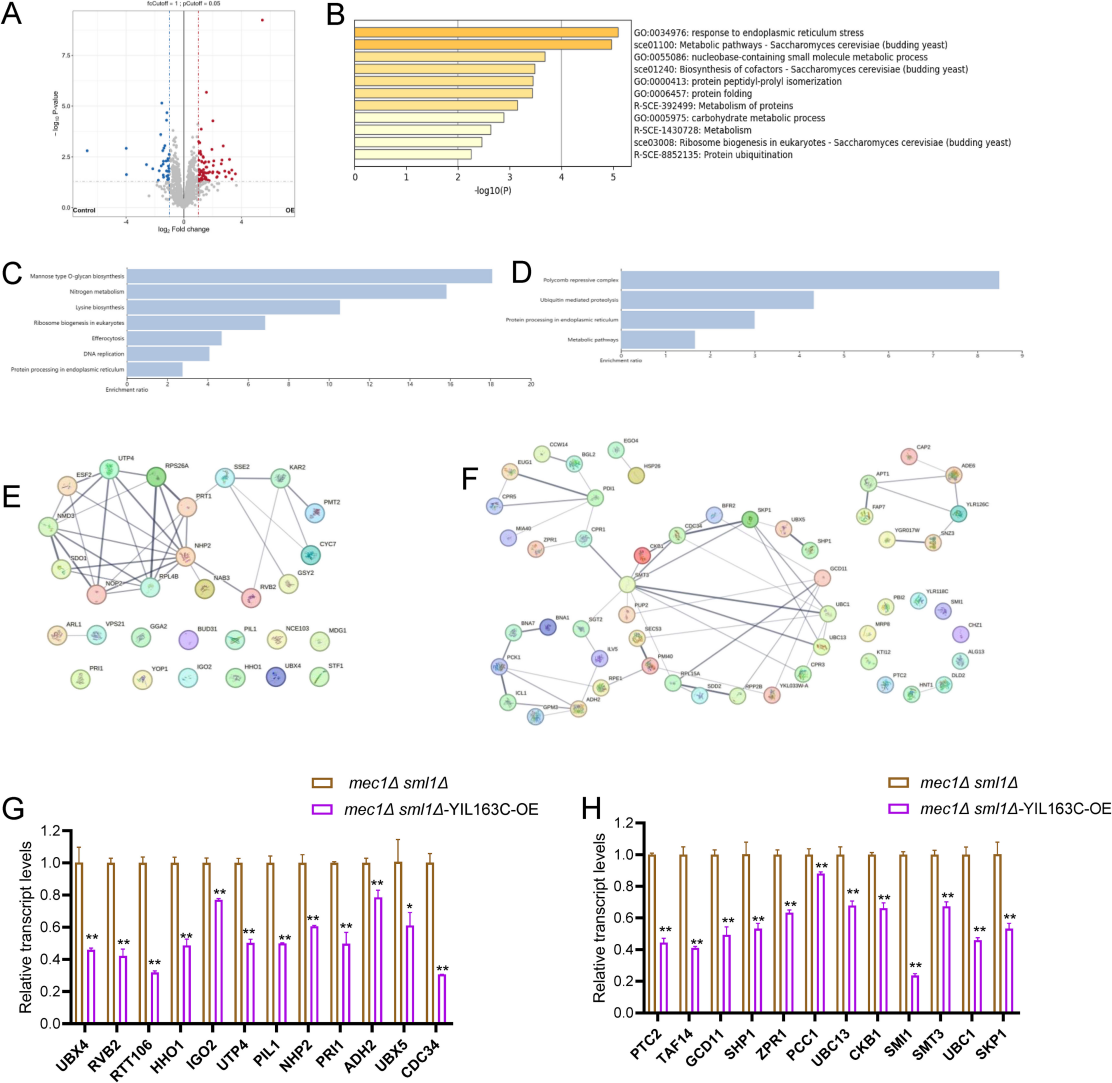
The impact of YIL163C overexpression on the proteome of *mec1Δ sml1Δ* cells during HU-induced DDR. **(A)** Volcano plot displaying the distribution of differentially expressed proteins in *mec1Δ sml1Δ* cells overexpressing YIL163C after HU treatment. Each dot represents a protein. Red dots indicate significantly upregulated proteins (fold change > 2, *p* < 0.05), blue dots represent significantly downregulated proteins (fold change > 2, *p* < 0.05), and gray dots correspond to non-differentially expressed proteins. **(B)** GO clustering analysis of the differentially expressed proteins. **(C,D)** KEGG pathway enrichment analysis of downregulated **(C)** and upregulated **(D)** proteins, respectively. **(E,F)** PPI network analysis of downregulated **(E)** and upregulated **(F)** proteins. **(G,H)** qPCR results of transcriptional changes in DDR-related proteins following HU treatment induced by YIL163C overexpression in *mec1Δ sml1Δ*. Data represent mean ± SD. *p < 0.05; **p < 0.01 (Student’s *t* test).

To examine how YIL163C overexpression influences proteins that reverse the replication stress sensitivity of *mec1Δ sml1Δ* cells, we conducted untargeted global mass spectrometry (MS)-based proteomics to analyze the protein response of YIL163C overexpression (*mec1Δ sml1Δ*-YIL163C-OE) under HU treatment. Our results revealed that YIL163C overexpression during replication stress led to notable changes in the cellular proteome, with 69 proteins being upregulated and 33 downregulated (FDR < 0.05, fold change > 2), compared to *mec1Δ sml1Δ* cells carrying an empty vector (Figure 2A; Supplementary Table S1). These differentially expressed proteins were implicated in ER-associated degradation, ER stress response, proteasomal protein catabolism, protein folding, and proteasome-mediated ubiquitin-dependent protein catabolic processes, as determined by Gene Ontology (GO) biological process annotation (Figure 2B). Subsequent Kyoto Encyclopedia of Genes and Genomes (KEGG) pathway analysis was performed on both upregulated and downregulated proteins. Downregulated proteins were primarily enriched in pathways including mannose-type O-glycan biosynthesis, nitrogen metabolism, lysine biosynthesis, ribosome biogenesis in eukaryotes, efferocytosis, and DNA replication (Figure 2C). On the other hand, upregulated proteins were significantly enriched in the polycomb repressive complex, ubiquitin-mediated proteolysis, protein processing in the ER, and various metabolic pathways (Figure 2D). Many of these proteins have been previously implicated in cellular responses to DDR (Table 4), suggesting that YIL163C overexpression may enhance tolerance to HU by promoting ribosome function and facilitating the ubiquitin-mediated degradation of aberrant proteins.

**Table 4:** Overexpression of YIL163C leads to changes in the expression of numerous proteins involved in the DDR response.

Protein	Uniprot ID	Fold change (Log2)	References
UBX4	P54730	−2.59	Chien and Chen (2013)
RVB2	Q12464	−1.77	Jha and Dutta (2009)
RTT106	P40161	−1.61	Clemente-Ruiz et al. (2011)
HHO1	P53551	−1.57	Hashimoto et al. (2007)
IGO2	Q9P305	−1.53	Juanes et al. (2013)
UTP4	Q06679	−1.41	Freed and Baserga (2010)
PIL1	P53252	−1.20	Pal et al. (2022)
NHP2	P32495	−1.08	Raghunandan et al. (2021)
PRI1	P10363	−1.07	Marini et al. (1997)
ADH2	P00331	1.07	Simpson-Lavy et al. (2015)
UBX5	Q06682	1.08	Noireterre et al. (2023)
CDC34	P14682	1.13	Silver et al. (1992)
PTC2	P39966	1.14	Leroy et al. (2003)
TAF14	P35189	1.20	Shanle et al. (2015)
GCD11	P32481	1.24	Lao et al. (2018)
SHP1	P34223	1.29	Hu et al. (2012)
ZPR1	P53303	1.35	Gangwani (2006)
PCC1	Q3E833	1.41	He et al. (2019)
UBC13	P52490	1.43	Brusky et al. (2000)
CKB1	P43639	1.54	Cheung et al. (2005)
SMI1	P32566	1.68	Hong and Huh (2021)
SMT3	Q12306	1.76	Soustelle et al. (2004)
UBC1	P21734	2.02	Arsenault et al. (2021)
SKP1	P52286	3.13	Thompson et al. (2022)

To further analyze the protein–protein interactions (PPI) and identify key network hubs among the differentially expressed proteins in *mec1Δ sml1Δ*-YIL163C-OE cells, we utilized the STRING database (Szklarczyk et al., 2019), the PPI network analysis revealed significant clusters (Figures 2E,F), with downregulated proteins predominantly involved in ribosome biogenesis (e.g., SDO1, NMD3, RPS26A, RPL48B), ER functions (e.g., KAR2, PMT2), and rRNA processing (e.g., NOP2, ESF2, UDP4, NHP2). Meanwhile, upregulated proteins aligned with the KEGG enrichment results, with hubs centered around transcriptional regulation (CKB1, SKP1), cell wall maintenance (CCW14, BGL2), post-translational modifications (EUG1, PDI1, CPR5, CPR1, ZPR1, MIA40), and DNA replication (e.g., APT1, FAP7, ADE6). Additionally, significant interactions were observed within the anaphase-promoting complex (APC)-mediated proteolysis network (e.g., SKP1, CDC34, UBC1, UBC13, SMT3, PUP2, UBX5, SHP1).

To validate the protein expression results, qPCR was utilized to examine the transcriptional levels of DDR-related differentially expressed proteins. It was observed that, following HU treatment, the transcriptional levels of all DDR-related differentially expressed proteins induced by YIL163C overexpression were significantly downregulated (Figures 2G,H). This suggests that the interplay between transcriptional and post-transcriptional regulation mediated by YIL163C may provide a sophisticated control mechanism for the DDR pathway.

### 3.3 Effects of YIL163C on the protein phosphorylation profiles

Post-translational modifications, particularly phosphorylation, are essential strategies for cells to respond quickly to environmental changes. Phosphorylation events are highly specific, reversible, and dynamic. Many known signal transduction events in the DDR are mediated by highly conserved checkpoint kinases. In *S. cerevisiae*, the S-phase checkpoint is regulated by the serine/threonine kinases Mec1 and Tel1 (Zeman and Cimprich, 2014). These kinases enhance DNA repair by phosphorylating downstream effectors and induce cell cycle delay, providing cells with more time to cope with elevated doses of DNA damage. Many downstream DNA repair proteins are phosphorylated during checkpoint activation, including those involved in post-replication repair (PRR), homologous recombination (HR), DNA replication, repair, histone modification, and chromatin remodeling (Bastos de Oliveira et al., 2015; Chen et al., 2010; Smolka et al., 2007). To more comprehensively characterize the signaling event of YIL163C overexpression in response to DDR stress, we performed phosphorylated proteomic analysis of *mec1Δ sml1Δ-YIL163C-OE* or *mec1Δ sml1Δ-EV* cells exposed to 10 mM HU for 2 h.

**Figure 3:**
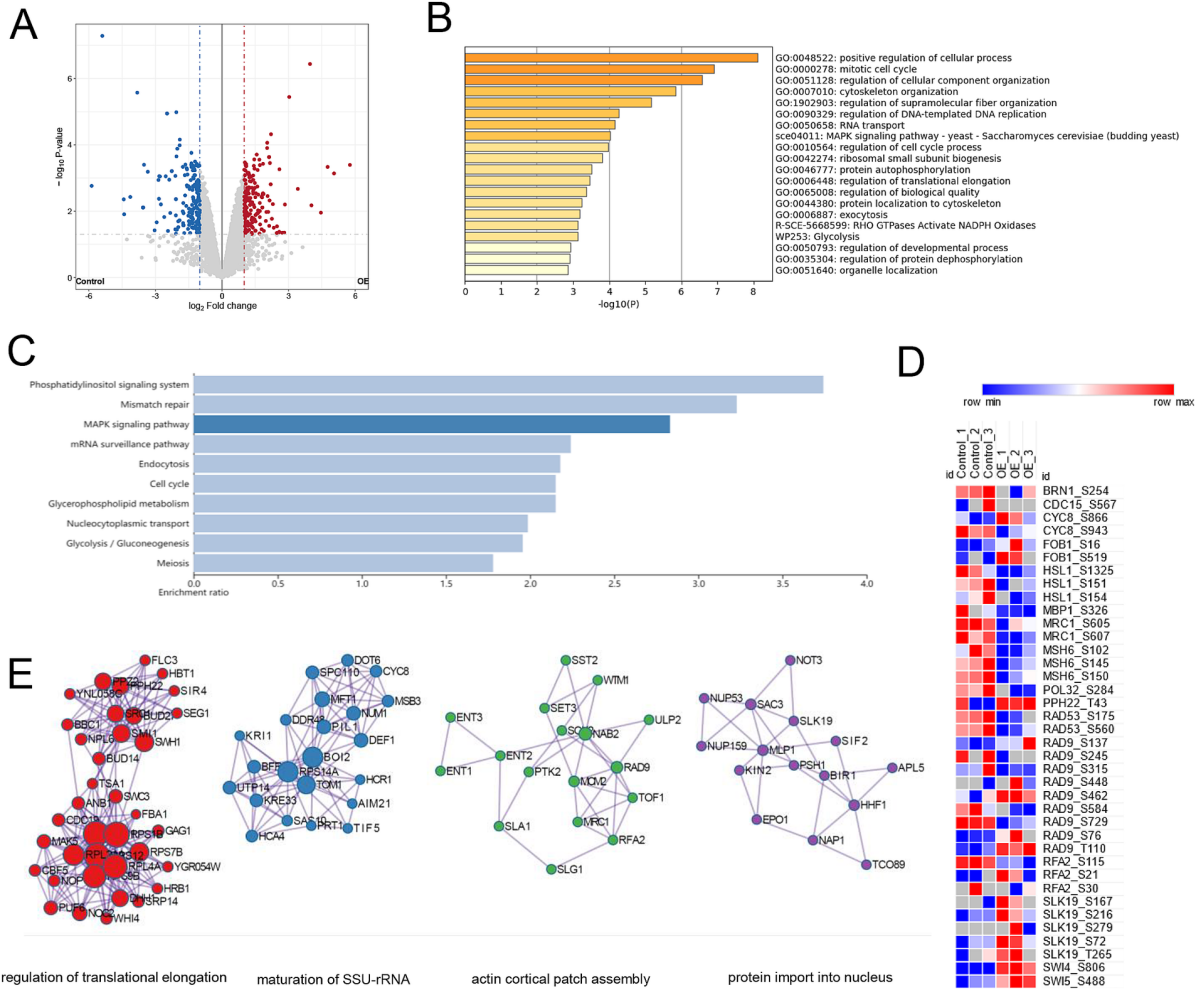
Phosphoproteomic analysis of *mec1Δ sml1Δ* cells overexpressing YIL163C during HU-induced replication stress. **(A)** Volcano plot illustrating the phosphorylation sites that exhibited significant changes in abundance (fold change ≥ 2, *p* < 0.05) in response to YIL163C overexpression in *mec1Δ sml1Δ* cells. **(B)** GO biological process enrichment analysis of the 267 differentially phosphorylated proteins. **(C)** KEGG pathway enrichment analysis of the differentially phosphorylated proteins. **(D)** Heatmap representing phosphorylation changes in key proteins involved in the DDR and DNA replication/repair. **(E)** PPI network analysis of the phosphorylated proteins.

For the phosphoproteomic analysis, a total of 5,525 phosphosites were confidently assigned to specific residues (with a Best Localization Probability > 0.75). Based on relative quantification, we identified 351 phosphopeptides corresponding to 267 proteins, each showing at least a two-fold change in abundance (*p* < 0.05) (Supplementary Table S2). The modifications were predominantly found on serine residues (278 phosphosites), with additional modifications occurring on threonine (71 sites) and tyrosine (2 sites) residues. In terms of directional changes within the *mec1Δ sml1Δ-EV* strain, 178 phosphopeptides displayed an increase in abundance following YIL163C overexpression, while 173 phosphopeptides showed a decrease (Figure 3A). The 267 proteins exhibiting altered phosphorylation in response to YIL163C overexpression were strongly associated with key GO biological processes, such as the positive regulation of cellular processes, the mitotic cell cycle, organization of cellular components, cytoskeleton organization, and DNA-templated replication. Additionally, other significant processes included RNA transport, MAPK signaling pathways, regulation of the cell cycle, ribosomal subunit biogenesis, and protein autophosphorylation (Figure 3B). KEGG pathway enrichment analysis further revealed that YIL163C overexpression primarily affected nuclear-related pathways, including the phosphatidylinositol signaling system, mismatch repair, mRNA surveillance, endocytosis, and the cell cycle (Figure 3C). Importantly, several differentially phosphorylated proteins involved in DNA replication and repair were identified, including MSH6, POL32, RFA2, BRN1, CDC15, CYC8, FOB1, HSL1, MBP1, MRC1, PPH22, RAD9, SLK19, SWI4, SWI5, and RAD53 (Figure 3D). These proteins are central to the DDR and other crucial cellular processes.

A PPI network analysis of these differentially phosphorylated proteins (Figure 3E) highlighted key nodes involving regulation of translational elongation, maturation of SSU-rRNA, actin cortical patch assembly, and protein import into nucleus. Notably, proteins such as RPL40A, RPS12, and RPS1B formed the core of the ribosomal biogenesis sub-network. These findings provide compelling evidence of a functional relationship between YIL163C and DDR-associated signaling pathways, suggesting that YIL163C modulates both DNA repair processes and overall genomic stability through specific phosphoprotein interactions.

### 3.4 YIL163C is involved in antifungal drug susceptibility

**Figure 4:**
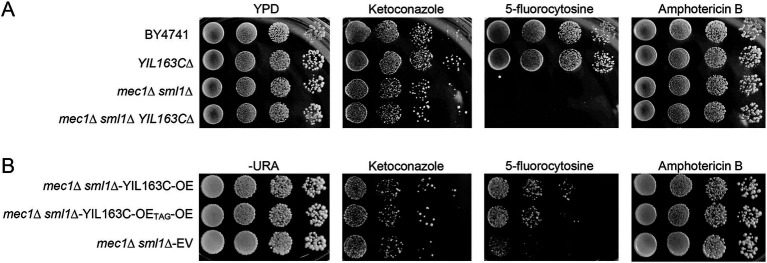
YIL163C overexpression reduces the sensitivity of *mec1Δ sml1Δ* to 5-fluorocytosine. Each *S. cerevisiae* strain was cultured in liquid YPD medium at 30°C, 10-fold serially diluted, spotted (5 uL) on YPD **(A)** or SC-URA **(B)** plate containing the indicated concentration of antifungal drugs (ketoconazole, 4 μg/mL; amphotericin B, 4 μg/mL; 5-fluorocytosine, 16 μg/mL), and further incubated at 30°C for 1–3 days. Plates were photographed daily.

To assess the impact of YIL163C on antifungal drug sensitivity, we investigated its overexpression and deletion in *S. cerevisiae* under HU stress. Our results revealed that YIL163C deletion did not affect drug susceptibility in wild-type strains (Figure 4A). The *mec1Δ sml1Δ* strain exhibits a hypersensitive phenotype to 5-fluorocytosine, but the deletion of YIL163C does not affect its sensitivity to 5-fluorocytosine in the *mec1Δ sml1Δ* background. Overexpression of YIL163C in the *mec1Δ sml1Δ* background reversed this hypersensitivity (Figure 4B). However, YIL163C did not alter sensitivity to other antifungal agents like ketoconazole or amphotericin B, suggesting its specific role in regulating DNA synthesis pathways targeted by 5-fluorocytosine. This aligns with studies showing that 5-fluorocytosine resistance in yeast involves DNA and RNA synthesis inhibition, and any disruption in DNA repair pathways can exacerbate sensitivity to this drug (Billmyre et al., 2020; Costa et al., 2015; Jung et al., 2019). Therefore, this result further demonstrates that YIL163C regulates the DDR pathway and influences the cell’s ability to respond to 5-FC-induced stress.

## 4 Discussion

This study demonstrates that YIL163C plays a role in rescuing the lethality of yeast cells lacking the Mec1 or Rad53 kinases, which are central to the DDR in *S. cerevisiae*. The successful rescue of *mec1Δ and rad53Δ* mutants by YIL163C overexpression highlights the emerging importance of lncRNAs in stress adaptation and genomic stability. Traditionally, DDR has been associated with protein-mediated mechanisms; however, our results introduce an additional layer of regulation through lncRNAs, particularly under replication stress conditions caused by genotoxic agents such as HU. The hypothesis that YIL163C functions primarily as a non-coding RNA was supported by introducing a stop codon mutation (TAG) to disrupt protein-coding potential. However, this does not entirely rule out the possibility of residual small peptide production or translational regulation effects. Even with a stop codon mutation, alternative translation initiation sites downstream of the mutation or translational read-through events could allow the production of small peptides. The observed increase survivability in YIL163C overexpression in response to HU suggests that it may play a contributory role in the Mec1-Rad53 pathway, potentially enhancing the cell’s ability to manage replication stress, as indicated by the effects of its overexpression. However, further experiments are required to determine its necessity under physiological conditions. The findings align with other studies that report lncRNAs participating in DDR and repair pathways. For example, lncRNA DDSR1 interacts with proteins like BRCA1 to enhance DSB repair via HR (Sharma et al., 2015). Similarly, lncRNA Gadd7 has been shown to modulate checkpoint activity by interacting with key regulatory proteins during the G1/S transition (Liu et al., 2012). These studies support the idea that lncRNAs function beyond gene transcription regulation, directly participating in stress responses and ensuring genome integrity under adverse conditions.

The proteomic analysis reveals that YIL163C modulates the abundance of numerous proteins involved in ribosome biogenesis, ubiquitin-mediated proteolysis, and DNA replication. These processes are essential for cell survival under replication stress, as they ensure proper protein folding, degradation of damaged proteins, and replenishment of dNTP pools required for DNA repair (Rudd et al., 2016). The enrichment of proteins involved in post-replication repair (PRR) and homologous HR suggests that YIL163C enhances the cellular machinery responsible for managing stalled replication forks and DNA lesions. The intriguing observation is that a subset of DDR-associated genes exhibited divergent trends between transcriptional regulation and translational output. The interplay between transcriptional and post-transcriptional regulation by YIL163C offers a sophisticated control mechanism for the DDR pathway. Whether this occurs through translational upregulation or protein stabilization remains to be determined, but both possibilities underscore YIL163C’s broader regulatory scope beyond transcription. As an RNA molecule, YIL163C probable influence the translational machinery directly. For instance, it might bind to ribosomes or translation factors, modulating the translation efficiency of specific mRNAs. Such interactions could either enhance or suppress protein synthesis of target genes, independent of changes in their mRNA levels. To dissect this dual regulatory mechanism, further studies are essential. Interestingly, the study identifies a bias toward phosphorylation events on serine and threonine residues, particularly on proteins associated with DNA replication and chromatin organization. This indicates that YIL163C influences post-translational modifications crucial for coordinating DDR processes. The phosphorylation patterns suggest a role for YIL163C in dynamically regulating checkpoint kinases and DNA repair enzymes, facilitating a rapid response to DNA damage.

A noteworthy aspect of the study is the functional interaction between YIL163C and known DDR inhibitors like Sml1. While deleting *SML1* alleviates the lethality of *mec1Δ* mutants by increasing RNR activity and dNTP levels (Zhao et al., 1998), YIL163C overexpression restores cell viability even in the absence of *SML1* suppression. This finding suggests that YIL163C operates through mechanisms distinct from traditional RNR regulation, potentially involving alternative pathways for nucleotide metabolism or stress response. Additionally, the observed changes in protein networks involved in ER stress response and ubiquitination function further emphasize the multifaceted role of YIL163C. The interplay between ER stress response and ubiquitination plays a significant role in the DDR, as these processes collectively support cellular recover under genotoxic stress. The ER stress response, through the Unfolded Protein Response (UPR), mitigates the accumulation of misfolded proteins and redirects resources toward DNA repair and cellular homeostasis under stress (Qu et al., 2021). Ubiquitination, via the ubiquitin-proteasome system (UPS), tags damaged or misfolded proteins for degradation, preventing cellular dysfunction and sustaining the DDR (Su et al., 2020). This process, including modifications such as UFMylation, which is involved in ER stress, has been shown to stabilize key DDR components under stress, helping cells effectively respond to DNA damage (Wang et al., 2023). By enhancing proteostasis, YIL163C likely mitigates the detrimental effects of stalled replication forks and DNA lesions, promoting cell survival under prolonged stress (Rickman and Smogorzewska, 2019).

The study’s investigation into antifungal drug sensitivity reveals that YIL163C modulates susceptibility to 5-fluorocytosine, a drug that targets DNA synthesis. YIL163C overexpression rescues the hypersensitivity phenotype of *mec1Δ sml1Δ* mutants to 5-fluorocytosine. This suggests that YIL163C enhances DNA repair pathways or nucleotide synthesis during replication stress, making it an important modulator of drug response. However, no significant effects were observed with other antifungal drugs like ketoconazole or amphotericin B, indicating that YIL163C’s influence may be specific to pathways linked to DNA metabolism. These findings underscore the potential of targeting lncRNA-mediated pathways in fungal pathogens to improve antifungal therapies. Developing strategies to modulate lncRNA activity could enhance the efficacy of DNA-targeting drugs and reduce resistance, providing new avenues for treating fungal infections.

While the study provides valuable insights into the role of YIL163C, several questions remain unanswered. First, the exact molecular mechanism by which YIL163C interacts with the Mec1-Rad53 pathway needs further elucidation. Identifying direct protein or RNA partners of YIL163C would provide a clearer picture of how it regulates DDR processes. Additionally, it would be interesting to explore whether YIL163C plays similar roles in other stress conditions, such as oxidative stress or nutrient deprivation, to determine whether its function extends beyond replication stress. Moreover, the study raises intriguing questions about the evolutionary conservation of YIL163C. Given the growing evidence of lncRNA involvement in mammalian DDR pathways, future research could investigate whether homologs or functional analogs of YIL163C exist in higher eukaryotes (Su et al., 2018). Understanding the cross-species conservation of these mechanisms would provide new insights into the broader role of lncRNAs in maintaining genomic stability.

## 5 Conclusion

In summary, this study identifies YIL163C as an important regulator of DRC and a player in the response to replication stress in yeast. By modulating protein abundance and phosphorylation, YIL163C ensures efficient DNA repair and cell cycle progression under stress conditions. Its ability to rescue the lethality of *mec1Δ rad53Δ* mutants highlights its potential as a novel effector in the Mec1-Rad53 signaling cascade. Furthermore, the findings on antifungal drug susceptibility suggest that YIL163C could serve as a valuable target for enhancing the effectiveness of DNA-targeting antifungal therapies. Future studies should focus on uncovering the precise molecular interactions involving YIL163C, exploring its role under various stress conditions, and investigating its conservation across different organisms. Overall, this research contributes to our understanding of the expanding role of lncRNAs in genomic stability and stress adaptation, paving the way for new therapeutic strategies in both fungal pathogens and human diseases involving genomic instability.

## Data Availability

The mass spectrometry data presented in the study are deposited in the ProteomeXchange repository, accession number PXD056715 (http://proteomecentral.proteomexchange.org/cgi/PXD056715).

## Supplementary material

SUPPLEMENTARY TABLE S1 The list of all differentially expressed proteins detected after YIL163C overexpression.

SUPPLEMENTARY TABLE S2 The list of all phosphorylation sites with differential phosphorylation detected in the phosphoproteome.
