# Supplementary figures and images for: A novel lncRNA YIL163C enhances genomic stability and antifungal resistance via the DNA damage response in Saccharomyces cerevisiae
Source: Front Microbiol. 2025 May 1;16:1571797. doi: 10.3389/fmicb.2025.1571797 (PMC12078222; doi:10.3389/fmicb.2025.1571797)

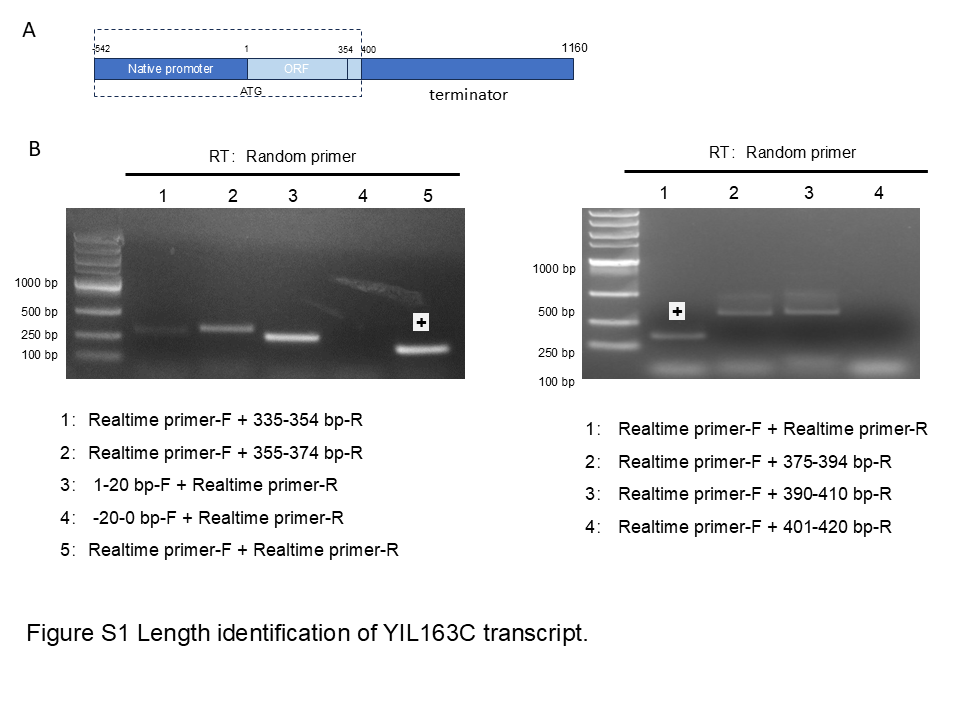

Supplement: Supplementary file 3 [file Image_1.tif]

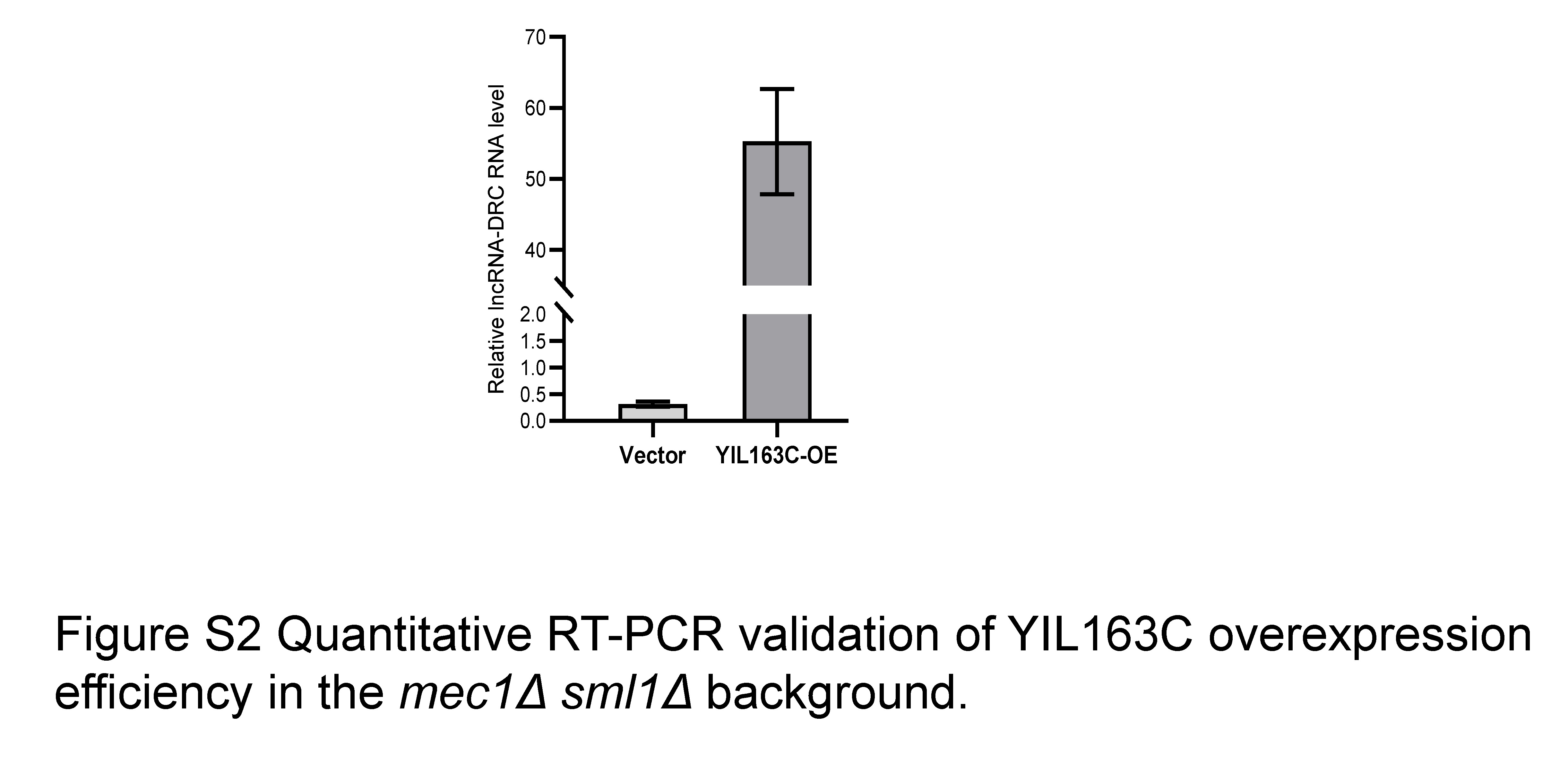

Supplement: Supplementary file 4 [file Image_2.jpeg]

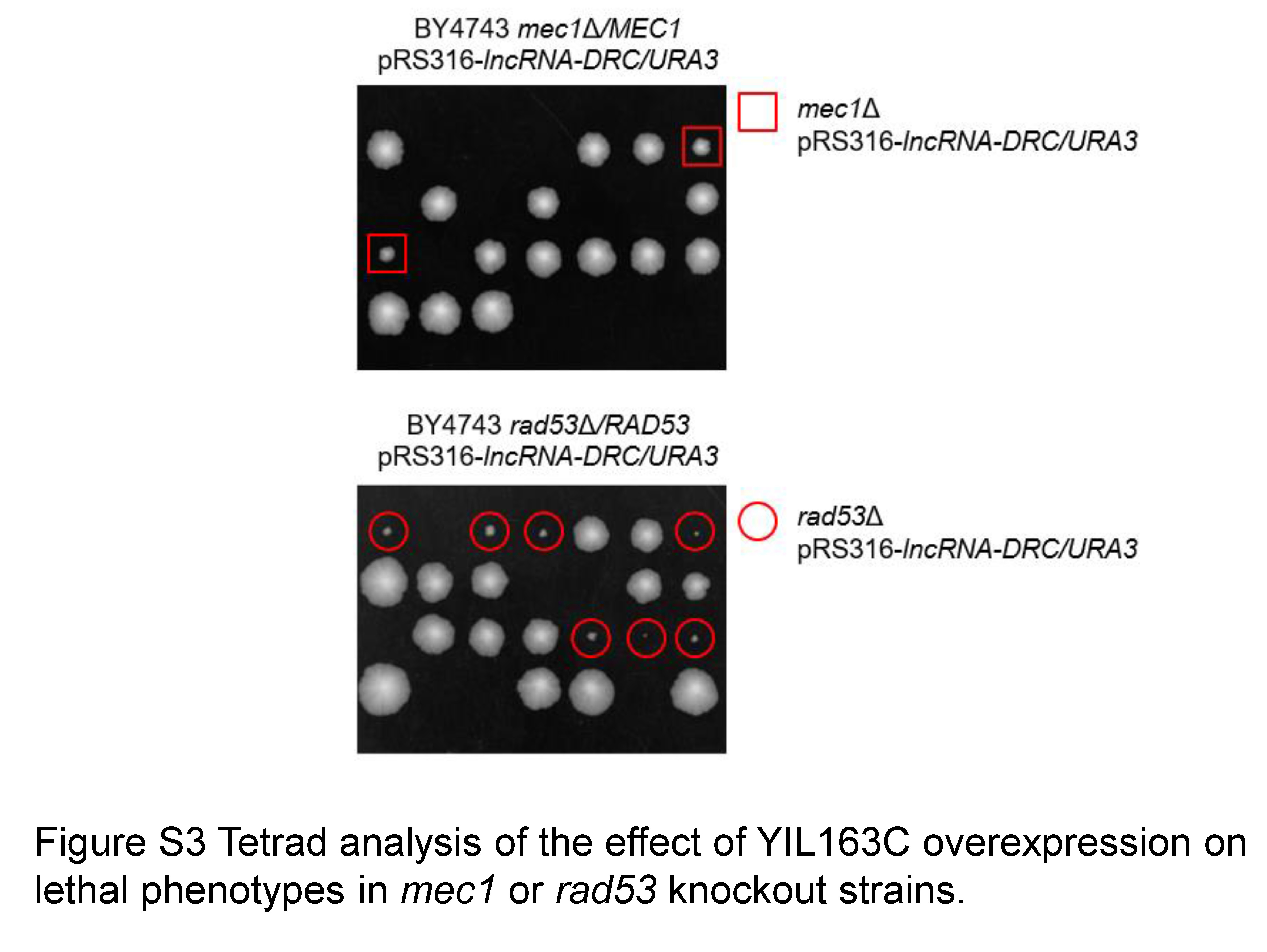

Supplement: Supplementary file 5 [file Image_3.jpeg]
